# Multisystem Involvement of Hydatid Disease

**DOI:** 10.4269/ajtmh.17-0995

**Published:** 2018-06

**Authors:** Onur Taydas, Serhat Kaya, Hayri Ogul, Mecit Kantarci

**Affiliations:** 1 Department of Radiology, Erzincan University Mengücek Gazi Training and Research Hospital, Erzincan, Turkey; 2 Department of Radiology, Faculty of Medicine, Ataturk University, Erzurum, Turkey

**Figure 1.**
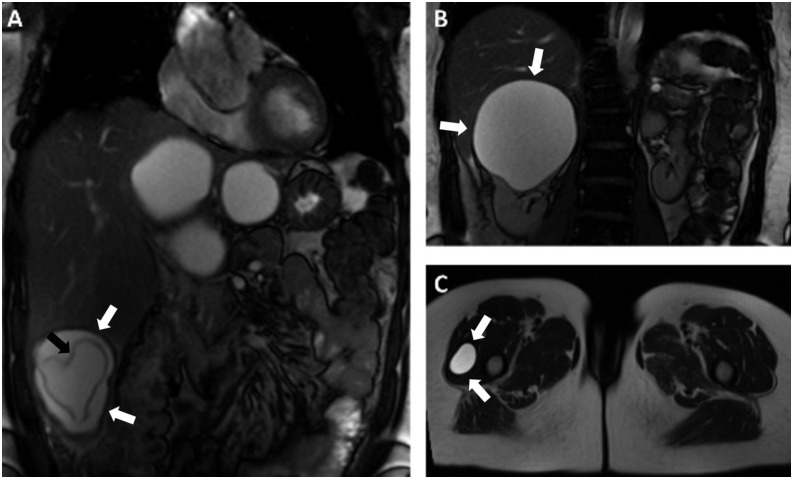
Coronal (**A** and **B**) and axial (**C**) T2-weighted images show hepatic, renal, and muscular hydatid cysts (white arrows). Note that a linear hypointense structure resembling an inner membrane is seen in the hepatic cyst (black arrow).

**Figure 2.**
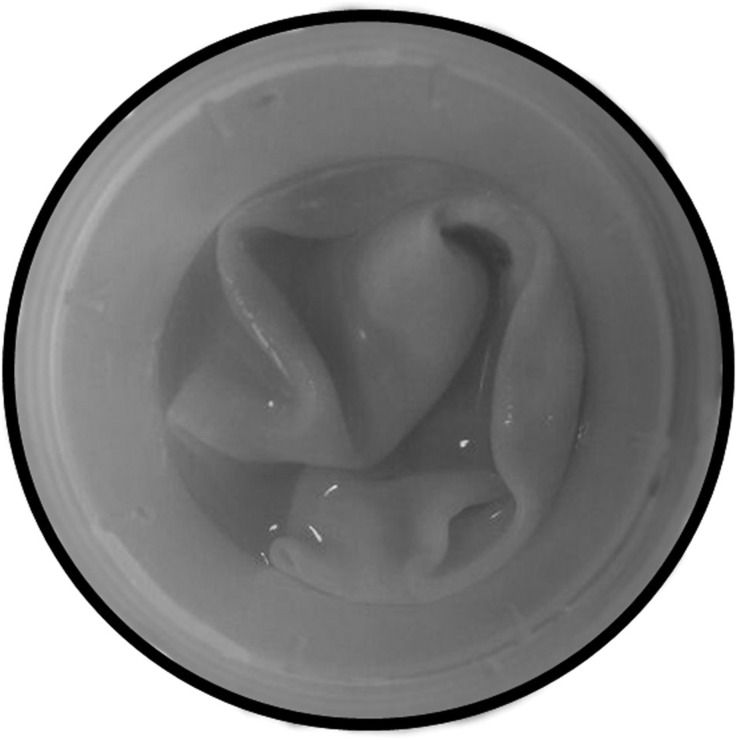
Postoperative photograph of the specimen showing the hydatid cyst.

A 54-year-old female who had no history of any chronic disease was admitted to our hospital with progressive abdominal pain and distension over the preceding 6 months. She had no history of vomiting, nausea, abdominal trauma, weight loss, or jaundice and she did not report any similar episode in the past. Physical examination revealed large round hypogastric and right hypochondriac masses that were palpable. There was also mild tenderness over her right hypochondriac region. Her body temperature and other vital signs were normal. Laboratory tests were also normal. Abdominopelvic magnetic resonance imaging (MRI) was performed for further investigation. Magnetic resonance imaging scans revealed hydatid cysts in the liver, right kidney, and right quadriceps femoris muscle (Figure 1). The hydatid cysts in the liver and right kidney were treated using the puncture, aspiration, injection, and respiration (PAIR) method. Surgical treatment of the hydatid cyst in the right quadriceps femoris muscle was performed (Figure 2). The pathology report confirmed echinococcal cysts.

Hydatid disease (HD) is an important public health problem, especially in countries where it is endemic. Clinical symptoms may occur in the later stages and are usually related to the mass effect. In most cases, physical examination and laboratory tests are not helpful for diagnosis. Therefore, radiology has a key role in the diagnosis of HD. The liver is the most common site of HD. The low-signal-intensity rim, which represents the outermost membrane of the cyst, is a characteristic finding on T2-weighted images for HD. Detached membranes are seen as linear hypointense structures on the MRI images.^1^ Renal HD is seen in 3% of all cases and is located mostly in the renal cortex. The findings of renal imaging are similar to those of hepatic HD. However, making a diagnosis to differentiate cystic tumors from abscesses is critical. Muscular involvement occurs in 2% of patients and can be confused with abscesses, chronic hematomas, and soft tissue tumors.^2^ Hydatid disease may be treated either surgically or by using a percutaneous approach. Percutaneous treatment consists of PAIR. In uncomplicated cysts, PAIR should be preferred because of the lower rates of complications.^3^
